# Resveratrol Treatment Delays Growth Plate Fusion and Improves Bone Growth in Female Rabbits

**DOI:** 10.1371/journal.pone.0067859

**Published:** 2013-06-28

**Authors:** Elham Karimian, Chen Tamm, Andrei S. Chagin, Karin Samuelsson, Kristín Rós Kjartansdóttir, Claes Ohlsson, Lars Sävendahl

**Affiliations:** 1 Pediatric Endocrinology Unit, Department of Women's and Children's Health, Karolinska Institutet, Stockholm, Sweden; 2 Division of Endocrinology, Department of Internal Medicine, Sahlgrenska University Hospital, Gothenburg, Sweden; 3 Department of Physiology and Pharmacology, Karolinska Institutet, Stockholm, Sweden; INSERM U1059/LBTO, Université Jean Monnet, France

## Abstract

*Trans*-resveratrol (RES), naturally produced by many plants, has a structure similar to synthetic estrogen diethylstilbestrol, but any effect on bone growth has not yet been clarified. Pre-pubertal ovary-intact New Zealand white rabbits received daily oral administration of either vehicle (control) or RES (200 mg/kg) until growth plate fusion occurred. Bone growth and growth plate size were longitudinally monitored by X-ray imaging, while at the endpoint, bone length was assessed by a digital caliper. In addition, pubertal ovariectomized (OVX) rabbits were treated with vehicle, RES or estradiol cypionate (positive control) for 7 or 10 weeks and fetal rat metatarsal bones were cultured *in vitro* with RES (0.03 µM–50 µM) and followed for up to 19 days. In ovary-intact rabbits, sixteen-week treatment with RES increased tibiae and vertebrae bone growth and subsequently improved final length. In OVX rabbits, RES delayed fusion of the distal tibia, distal femur and proximal tibia epiphyses and femur length and vertebral bone growth increased when compared with controls. Histomorphometrical analysis showed that RES-treated OVX rabbits had a wider distal femur growth plate, enlarged resting zone, increased number/size of hypertrophic chondrocytes, increased height of the hypertrophic zone, and suppressed chondrocyte expression of VEGF and laminin. In cultured fetal rat metatarsal bones, RES stimulated growth at 0.3 µM while at higher concentrations (10 μM and 50 μM) growth was inhibited. We conclude that RES has the potential to improve longitudinal bone growth. The effect was associated with a delay of growth plate fusion resulting in increased final length. These effects were accompanied by a profound suppression of VEGF and laminin expression suggesting that impairment of growth plate vascularization might be an underlying mechanism.

## Introduction

Linear bone growth is the consequence of chondrocyte proliferation, hypertrophy and terminal differentiation in the growth plates of the long bones. In the growth plate, immature cells lie toward the epiphysis, called the resting zone, with flat, more mature chondrocytes in the proliferating zone and large chondrocytes in the adjacent hypertrophic zone. At the end of puberty, longitudinal growth ceases with total replacement of avascular cartilage by highly vascularized bone, eventually resulting in epiphyseal fusion. Growth is influenced by many intrinsic and extrinsic factors such as heredity, genetic, illness and medications, nutrition and hormones. Growth disorder in the form of extreme short or tall stature is one of the most frequent reasons why a patient is referred for endocrinological assessments. In most cases there is no identifiable cause of their growth disorder and they are therefore are commonly labeled as having idiopathic short or tall stature. High-dose estrogen treatment has since the 1950s been used to reduce adult height in tall girls [1]. Conversely, inhibition of estrogen production by aromatase inhibitors (AIs) has more recently been shown to increase predicted adult height [2] and near final height in boys [3]. However, high-dose estrogen therapy appears to reduce fertility later in life [4], increases the risk for deep vein thrombosis [5]and, possibly, increases the risk for breast and gynecological cancers [6]. Similarly, treatment of pre-pubertal boys with AI for 6 months aiming to improve their final height seems not to be safe [7]. It has been reported that it causes an increase in bone resorption and bone formation [7]. Moreover, longer treatment of pre- or early pubertal idiopathic short stature males with an AI led to vertebral deformities [8]. To improve bone growth, GH therapy also is applicable only to a few disorders such as GH deficiency (GHD), Turner syndrome, idiopathic short stature, small for gestational age with failure to attain normal growth percentiles, Prader-Willi syndrome, chronic renal insufficiency and Noonan syndrome. Also it is worth mentioning that only children and adolescents with GHD have the greatest response to GH therapy. These findings emphasize the lack of suitable strategies to treat extreme short or tall adolescents while having fewer side effects.

*Trans*-resveratrol (3, 5, 4′-trihydroxystilbene), is a polyphenol naturally produced by a variety of plants such as peanuts, berries, skin of red grapes in response to stress, injuries and infections. It was isolated in 1963 from the roots of *polygonum cuspidatum* and described as medication for inflammation, carcinogenesis and cardiovascular diseases [9]. Its structure is similar to the potent and synthetic estrogen, diethylstilbestrol, suggesting that it might have some estrogenic activity. Scientific interest has grown continually since 1992 when some of the cardioprotective effects of red wine were postulated to RES [10] and later on, when it was first shown to prevent carcinogenesis by blocking tumor initiation, promotion and progression in mice [11]. RES has multiple mechanisms of action which might be related to all its beneficial effects [12]. In breast cancer cells, a significant decrease in extracellular level of vascular endothelial growth factor (VEGF) has been reported following RES treatment [13]. In rats, it has also been reported that heart expression of VEGF is elevated already after 24 hours of RES consumption [14]. To our knowledge, there is only one previous report in weanling rats demonstrating that 6 days of treatment with very low doses of RES (1–100 μg/day) had no significant effect on radial bone growth [15]. However, it has been reported that RES prevents progression of osteoarthritis in experimental osteoarthritis (OA) in rabbits [16] due to a decrease in chondrocyte apoptosis and nitric oxide production. Moreover, this chondro-protective effect of RES in articular cartilage of pigs was shown to be due to inhibition of accumulation of glycation end products in the joint cartilage [17]. These findings further support the possible effect of RES on growth plate cartilage. There is evidence showing that this compound is well tolerated even at relatively high doses in humans without having serious side effects [18], [19]. However, extremely high oral doses of RES (1 or 3 g/kg) have been reported to decrease food consumption and body weight gain while inducing renal failure in rats [20].

The aim of the present study was to evaluate the long-term effects of RES on growth plate cartilage, bone growth and growth plate fusion. To achieve this, we used different experimental models; pre-pubertal ovary-intact female rabbits, pubertal OVX rabbits [21] and an *ex vivo* model of cultured fetal rat metatarsal bones [22]. We chose rabbits to allow studies of the timing of growth plate fusion [23] which, in contrast to small rodents, normally occur at the time of sexual maturation, just like in humans.

## Material and Methods

### Materials

5-[(1E)-2-(4-Hydroxyphenyl)ethenyl]-1,3-benzenediol; 3,4′,5-Trihydroxy-*trans*-stilbene (resveratrol) for *in vitro* study, BSA and β-glycerophosphate were purchased from Sigma-Aldrich, Inc. (Steinheim, Germany). *Trans*-resvertrol for the *in vivo* study was purchased from MegaResveratrol, Danbury CT 06810, USA. Phosphate buffer saline (PBS), DMEM/F12 medium and gentamycin were from Invitrogen, Inc. (Paisley, UK).

### Animals

Experiment 1: Twenty four pre-pubertal, 12-week-old female New Zealand white rabbits (Linköping, Sweden) were divided to two groups after being matched for body weight; control (vehicle; n = 12) and *trans*-resveratrol (RES; n = 12). During 16 weeks, all animals were given a daily 2–3 ml administration of 7% ethanol/0.9% NaCl (control) or 200 mg/kg/day *trans*-resveratrol dissolved in 7% ethanol/0.9% NaCl given orally by syringe. We aimed to continue the experiment until growth plate fusion was achieved in all animals. After 16 weeks of study, the distal tibia, distal femur and proximal tibia growth plates were fused in all animals. In this set of experiment, two rabbits in the treatment group died due, we believe, to stress when anesthetized for x-ray examination.

Experiment 2: Similarly, thirty nine pubertal, 16-week-old, ovariectomized (OVX) rabbits were divided into three experimental groups; control (vehicle; n = 17), *trans*-resveratrol (RES; n = 17), and estradiol cypionate (E2; n = 5). The use of OVX animals enabled us to perform morphometric and mechanistic studies in a wide growth plate which undergoes fusion rather late after sexual maturation because of estrogen deficiency. The animals received the same dose and route of administration of RES and vehicle. An additional E2 group (positive control) received estradiol cypionate in sesame oil at a dosage of 70 μg/kg which was administered as a weekly intramuscular injection while the rest of the animals received the corresponding volume of sesame oil only (0.1–0.25 ml). We avoided mixing RES with their food because trans-resveratrol tends to oxidize quickly in the presence of light and/or oxygen. After 7 weeks, all animals in the E2 group and 5 animals each from the control and RES groups were sacrificed in order to perform morphometrical and immunohistochemical staining in an unfused growth plate. The treatment was continued for the remaining animals for an additional 3 weeks in order to study the effect of RES on the timing of growth plate fusion in different epiphyses.

Throughout the study, all rabbits received a soy bean–free diet (SD 573; Lantmännen Lantbruk, Kimstad, Sweden) and tap-water *ad libitum* and were subjected to a 12-h light/dark cycle. After death, induced by a high-dose of pentobarbital, blood and tissue samples were collected for subsequent analysis.

### Ethics statement

This study was performed in strict accordance with the recommendations regarding animal use at Karolinska Institutet and in Sweden. The experiments were approved by the Committee on the Ethics of Animal Experiments of the Northern Stockholm Area (Stockholms Norra Djurförsöksetiska Nämnd) and covered in full by the permits with numbers N245/07, N270/11 and N49/05. Efforts were made to avoid animal suffering while handling the animals with all related procedures for the current study.

### Organ culture of fetal rat metatarsal bones

Rudiments of metatarsal bones were collected from rat embryos at 20 days of gestation as previously described [24]. Briefly, the three middle metatarsals were dissected from the hind paw and, after removing soft tissues, transferred to 24-well plates, where they were cultured in 1 ml of phenol red-free DMEM/F12 medium supplemented with 0.2% BSA (endotoxin–free fraction V), 1 mM β-glycerophosphate, 0.05 mg ascorbic acid per ml and 20 μg gentamicin per ml at 37°C under a humidified atmosphere containing 5% CO2. The bones were co-cultured with either vehicle (dimethyl sulfoxide; DMSO) or RES (0.03 µM to 50 µM) dissolved in DMSO. The medium was changed every 2–3 days and bone length measured on the first day (designated as day zero) and after 2, 5, 7, 12 and 19 days using the Microimage^TM^ software ver. 4.0 (Olympus Optical Co., Hamburg, Germany). The increases in bone length from day 0 (the harvesting day) are presented as percentages.

### Evaluation of bone length and bone maturation

Radiographic images were taken at the start of the study and then every other week until the end of the study. The rabbits were anesthetized by an intramuscular injection of Ketamin (25 mg/kg) and Xylazine (5 mg/kg) prior to radiographic imaging. Settings used were: 50 kV and 2.5 mAs, at 104 cm distance. The right tibia was visualized in lateral projection with the rabbit in the prone position while the spine was displayed with the rabbit in a lateral position. The right tibia was measured from its proximal end to its distal end. The spinal length was measured from the cranial end of the second vertebrae to the caudal part of the fourth lumbar vertebrae (C2-L4). Growth values indicate increase in length from the start of the experiment. Due to technical problems, after 10 weeks of treatment we were not able to collect growth data in ovary-intact animals. Instead, bone length was measured by a digital caliper at the end point (16 weeks).

Bone maturation was studied in live animals (X-ray images) and in harvested femur and tibia after sacrifice. The growth plate was considered to be fused when 50% or more of the cartilage was substituted by bone bridges and data is presented as the percentage of animals with unfused growth plate. All measurements were performed by a blinded observer.

### Body weight, food intake and blood sampling

Food intake was measured daily and body weight weekly throughout the rabbit studies. Blood was collected from an ear vein every two weeks while being anesthetized for X-ray imaging. The blood was kept at room temperature for one hour to allow clotting and centrifuged at 3000 rpm for 15 minutes. Supernatants were separated and stored at −80°C for later analysis.

### Tissue collections

Right and left femur and tibia were fixated in 4% freshly prepared formaldehyde for 24 hours and then stored in 70% ethanol at +4°C until decalcification, paraffin embedment and sectioning. Uterus weight was measured at time of sacrifice to verify its responsiveness to E2 and any possible estrogenic activity of RES.

### Peripheral quantitative computed tomography (pQCT)

Tomographic measurements were performed using the Stratec XCT Research M (software version 5.4B; Norland Medical Systems Inc.) adapted specially for the examination of small bone specimens [25]. Parameters associated with trabecular and cortical bone structures for instance; trabecular bone mineral density, cortical content, cortical area, cortical thickness circumference, periosteal- and endosteal circumferences were evaluated as described previously [26].

### Quantitative histology of the growth plate and analysis of serum IGF-1

Sections of the femur growth plate were stained with Masson's trichrome and histological parameters were then quantified as described previously [27]. Briefly, the height of the growth plate, numbers of proliferative and hypertrophic chondrocytes per column, the resting zone area, proliferative and hypertrophic zone height and the size of the terminal hypertrophic chondrocytes were determined by analysis of digital images from the center two-thirds of the growth plate with the Microimage^TM^ software ver. 4.0 (Olympus Optical Co., Hamburg, Germany). The presented values are means of 24 measurements on each individual growth plate. Chondrocytes >7 µm were considered to be hypertrophic. All measurements were done blindly. Serum levels of IGF-1 were measured by a double-antibody IGF-binding protein-blocked commercial RIA kit (Mediagnost, Tubingen, Germany).

### Cell proliferation and apoptosis detection

Due to the low proliferative activity of epiphyseal chondrocytes, double injection of BrdU was performed. Therefore, rabbits received an intraperitoneal injection of BrdU (50 mg/kg) 2 and 16 hours prior to sacrifice and subsequent detection of BrdU-positive cells in the growth plate was achieved utilizing a cell proliferation kit (Amersham Biosciences, Buckinghamshire, UK), according to the manufacturer's protocol. The chondrocyte proliferation rate was expressed as the number of BrdU-positive cells per unit area in the growth plate.

Apoptotic chondrocytes were identified by terminal deoxynucleotidyl transferase (TdT)-mediated deoxy-UTP nick end labeling (TUNEL) immunohistochemistry applying the TdT-FragEL™ DNA fragmentation kit (Oncogene Research, Boston, MA) as previously detailed by us [28].

### Tartrate-resistant acid phosphatase (TRAP) staining for osteoclast detection

Formaldehyde fixed, decalcified and paraffin-embedded rabbit epiphyses were sectioned and stained for tartrate-resistant acid phosphatase (TRAP) using an Acid Phosphatase Leukocyte Kit (Sigma-Aldrich, 387A, Germany) according to the manufacture's protocol. Osteoclasts were identified as TRAP-positive multinucleated cells containing at least 3 or more nuclei at the chondro-osseous junction by light microscopy.

### Immunostaining of angiogenesis factors; Vascular Endothelial Growth Factor (VEGF) and laminin

Immuno-detection of VEGF and laminin were carried out according to a standard protocol for immunohistochemistry with some modifications. After deparaffinization in xylene (2×15 min) and rehydration in descending grade of ethanol, antigen retrieval was achieved by incubating the slides at 85°C in a sodium citrate buffer for 30 min in a water bath or by incubating with 0.05% tripsin EDTA.for 15 min at 37°C. Subsequently, endogenous peroxidase activity was inhibited and nonspecific antibody binding eliminated with 3% of serum in 1% BSA in PBS at room temperature for 70 min prior to the addition of the primary antibody. Sections (4–5 µm) were incubated overnight with a mouse monoclonal antibody against VEGF (NeoMarkers, Runcorn, UK) or rabbit polyclonal antibody against laminin (Abdserotec, Bio-Rad laboratories, Inc) diluted 1∶50 and 1∶100, respectively. Sections were then incubated with rabbit anti-mouse IgG 1∶300, (Dako, Glostrup, Denmark) or goat anti-rabbit IgG 1∶400, (Dako, Glostrup, Denmark) followed by a signal enhancer, the avidin-biotin complex (Vectastain ABC kit, PK-4001; Vector Laboratories, Burlingame, CA). After detection of positive chondrocytes by 2% diaminobenzidine, slides were counterstained with Alcian blue/hematoxyline, dehydrated and mounted. In some sections, the primary antibody was omitted or incubated with rabbit immunoglobulin (Dako, A/S, Denmark) at the same concentration as the primary antibody serving as negative controls. In addition, as a positive control; human placenta was used for VEGF and rabbit placenta for laminin.

An attempt to study other important local regulators of the longitudinal bone growth i.e. PTHrP (Oncogene Science, Cambridge, MA, USA), Collagen X (Quartett (Berlin, Germany) and active caspase 3 IHC was also made. However, due to the limited suitability of available antibodies for formaldehyde fixed tissues for immunohistochemical studies in rabbit, it was not successful.

### Statistical analyses

All data are expressed as means ± SEM. Differences between control and treated groups were evaluated with the One Way Analysis of Variance (ANOVA) followed by the Holm-Sidak post-hoc test and, for comparisons between two groups only, the t-test was applied. Treatment effects on growth plate fusion were evaluated by Kruskal-Wallis One Way ANOVA on Ranks. A p-value of less than 0.05 was considered statistically significant.

## Results

### Effects of RES on bone growth and final bone length in ovary-intact rabbits

**Figure 1:**
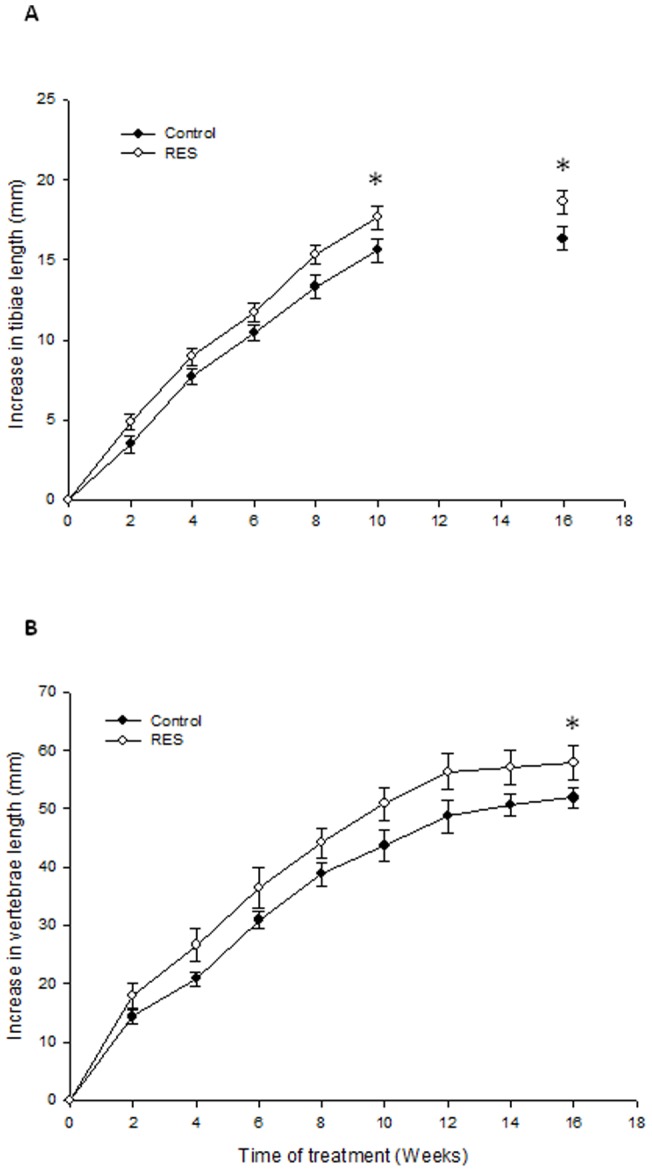
Mean tibia (A) and vertebrae (B) growth from treatment start in ovary-intact rabbits treated with resveratrol (RES) or vehicle (Control) for 16 weeks (n = 24). ^*^p<0.05 vs. control.

First, we followed tibiae and vertebrae (C2-L4 distance) growth by doing X-ray measurements. RES increased tibiae growth after eight weeks of treatment (Fig. 1A). This observation was further confirmed by digital caliper measurements performed upon bone dissection (tibia length 111.6±0.6 mm in the RES group vs. 109.5±0.6 mm in control; p<0.05). The C2-L4 distance increased in RES-treated animals early in the study and this distance was also longer at the end point when compared with control (Fig. 1B). Femur length was also increased in RES-treated animals, albeit not significantly (103±0.8 vs. 101.5±0.6 mm; p = 0.1).

### Effects of RES on bone growth and final bone length in OVX rabbits

**Figure 2:**
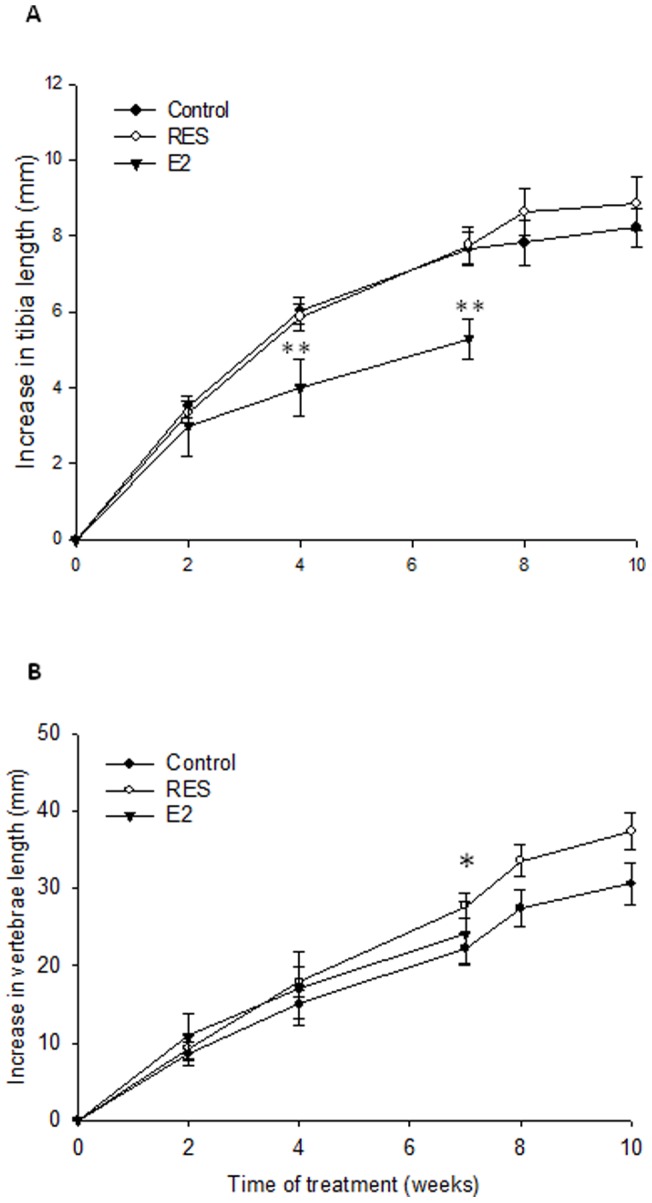
Effect of RES and E2 on tibiae (A) and vertebrae (B) growth in OVX rabbits. ^*^p<0.05 and ^**^p<0.01 vs. controls.

When bone length was measured by a digital caliper at the end, the femur was significantly longer in RES-treated animals (102.4±0.6 mm vs. 100.9±0.4 mm in controls; p<0.05) while the tibia length did not significantly differ between the RES group and controls (107.5±0.5 vs. 107.1±0.6 mm). When growth was followed by x-ray and compared to control rabbits, RES treatment did not affect tibiae growth at any of the time points while E2-treated animals showed a decrease in tibiae growth which was statistically significant already after 4 weeks of treatment (Fig. 2A).

The C2-L4 distance was significantly longer in RES treated rabbits, an effect seen already after 7 weeks, while in E2-treated animals it was not significantly affected when compared to controls (Fig. 2B). At end point, the C2-L4 distance was still longer in the RES group compared with controls, although not significantly (Fig. 2B).

### Effect of RES on the time of growth plate fusion in OVX rabbits

**Figure 3:**
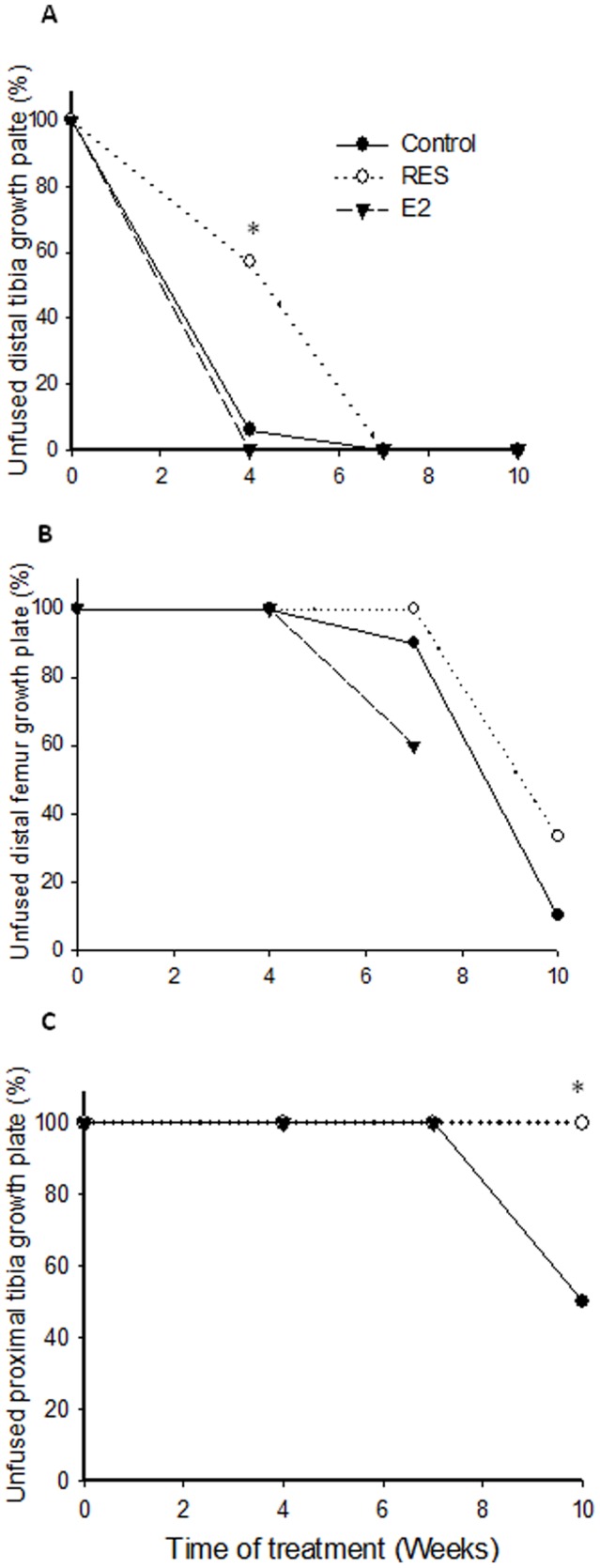
Percent of OVX rabbits with unfused growth plates; in the distal tibia (A), distal femur (B) and proximal tibia (C). Growth plates were considered to be fused if 50% or more of the growth plate cartilage was replaced by bone bridges. ^*^p<0.05 vs. control.

When assessed after 4 weeks treatment, the distal tibia growth plate was unfused in 57% of RES-treated (8 out of 14) animals while it was unfused in 6% of controls (1 out of 17) and fused in all animals in the E2 group (Fig. 3A; p<0.05 vs. control). At 7 weeks, the distal femur growth plate, known to undergo later fusion than the distal tibia growth plate, was unfused in all RES-treated animals while it was unfused in 90% of controls and in 60% of E2-treated animals (Fig. 3B). At this time point, the proximal tibia growth plate, known to undergo even later spontaneous fusion, was still open not only in all RES and control animals but also in all E2-treated rabbits (Fig. 3C). After 10 weeks, 33% of the animals in the RES group had unfused distal femur growth plates while only 10% of control animals had (Fig. 3B). At this time point, no proximal tibiae growth plate was fused in RES-treated animals while 50% were fused in controls (Fig. 3C, p<0.05). Thus, RES-treatment delayed the time of growth plate fusion in all studied growth plates.

### Effects of RES on body weight, food intake and serum IGF-I

In ovary-intact rabbits, 16-week RES treatment did not affect food intake (121±4.5 vs. 129±4.9 g/day in controls) or body weight (3615±91 vs. 3671±83 g in controls). In OVX rabbits, 7 weeks` treatment with RES or E2 did not affect food intake (139±3.8 and 126±4.0 g/day, respectively vs. 130±4.6 g/day in controls) or body weight (3139±122 and 3339±112 g, respectively vs. 3264±175 g in controls). Also after 10 weeks' treatment, RES had no effect on food intake (137±2.7 vs. 139±2.9 g in control) and body weight (3385±77 vs. 3411±72 g in controls). When analyzed after 7 weeks, serum IGF-I was similar in RES, E2 and control OVX rabbits (Table 1). Also after 10 weeks, serum IGF-I was similar in RES and E2 treated OVX rabbits (Table 1).

**Table 1:** Femur growth plate morphometry, osteoclasts number, uterus weight and serum IGF-I levels in ovariectomized rabbits^1^.

	Experimental groups		
	Control	RES	E2
Average area of resting zone (mm^2^)	0.11±0.02	0.26±0.05^**^	0.08±0.02
Proliferative zone height (µm2)	173±24	125±14	96±24
Hypertrophic zone height (µm2)	71±5	95±5^**^	51±14
Proliferative chondrocytes (cells/column)	5.2±0.9	4.5±0.6	3.2±1.3
Hypertrophic chondrocytes (cells/column)	3.3±0.1	5.0±0.1*	2.6±1.1
Average size of terminal hypertrophic chondrocytes (μm)	10.5±0.6	12.4±0.6*	10.0±0.6
Osteoclasts number (Number of TRAP-positive/growth plate)	22.4±3.2	15.2±4.9	6±1.6^**^
Serum IGF-I levels (ng/ml)			
7 wks treatment	191±11	203±23	208±16
10 wks treatment	140±8	122±7	
Uterus weight (g)			
7 wks treatment	0.6±0.1	0.5±0.1	18±2^***^
10 wks treatment	0.6±0.1	0.6±0.1	

1 Growth plate morphometry was performed after 7 weeks' treatment.

* p<0.05, ^**^p<0.01 and ^***^p<0.001 compared to vehicle-treated animals.

### Effects of RES on growth plate histology, chondrocyte proliferation and apoptosis in OVX rabbits

**Figure 4:**
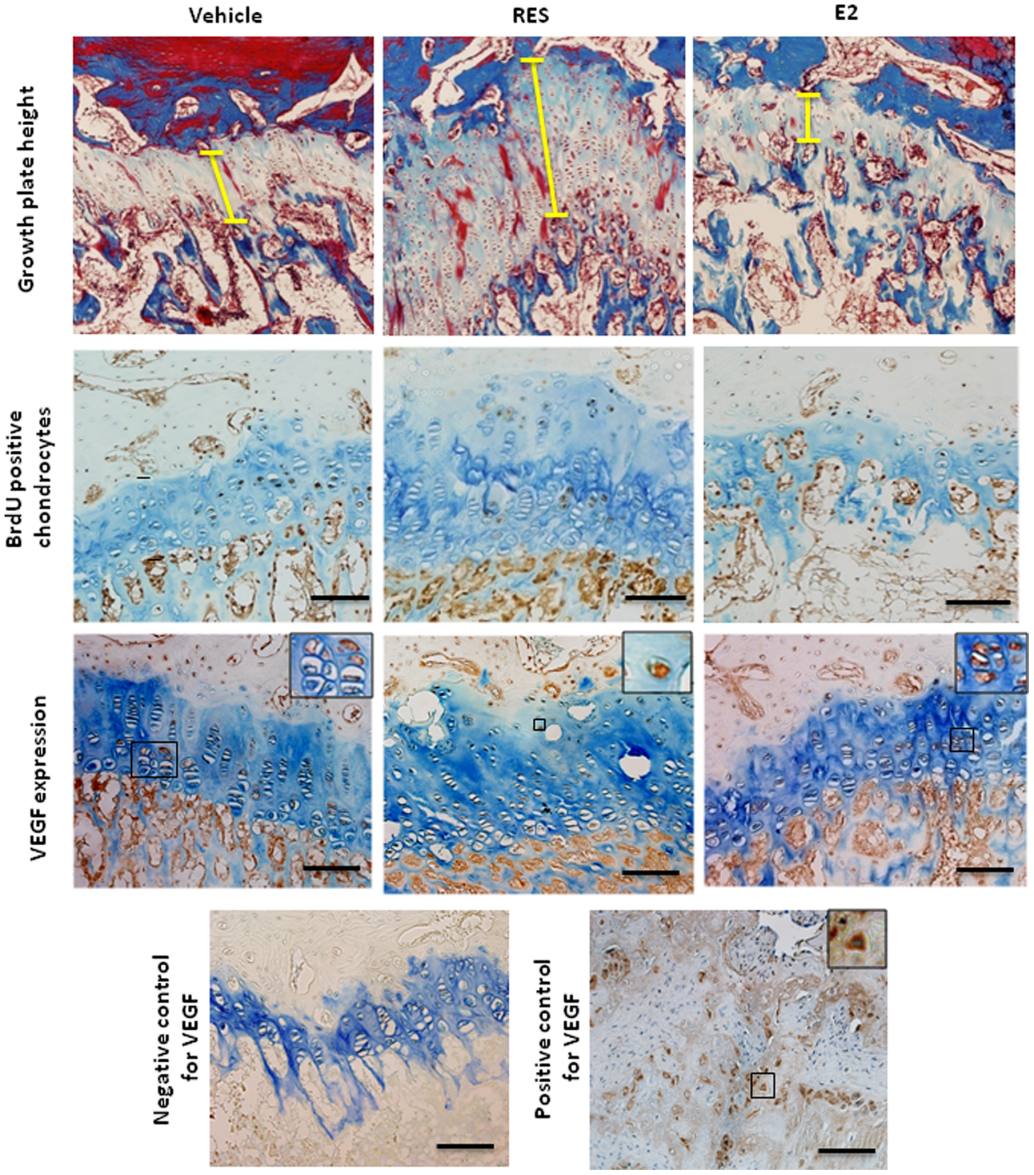
Representative images of the distal femur growth plate of OVX rabbits treated for 7 weeks with vehicle, RES or E2. Masson's trichrome stained growth plates with vertical bars depicting the height of the growth plates. Brown nuclear staining indicates proliferative chondrocytes (BrdU-positive). Expression of VEGF protein in the same growth plates of vehicle, RES- and E2-treated animals. No staining was observed when sections were incubated with rabbit immunoglobulin (negative control) while strong cytoplasmic and extracellular matrix stainings were observed when the primary VEGF antibody was applied to human placenta (positive control). Bar  = 200 µm.

To further identify the underlying mechanism behind the observed effects of RES, histomorphometrical analysis of the distal femur growth plates was performed after 7 weeks' of treatment. The height of this growth plate had then increased more than 2-fold in the RES group compared with controls (284±19 vs. 133±40 μm; p<0.01; Fig. 4). In contrast, E2-treated animals had narrower growth plates than controls, although this did not reach statistical significance (72±31 vs. 133±40 μm; p = 0.19, Fig. 4). A clear increase in resting zone area was observed in RES-treated animals when compared with controls, p<0.01 (Table 1). The numbers of proliferative chondrocytes per column and the height of the proliferative zones were not statistically different in animals treated with RES or E2 when compared with controls (Table 1). In contrast, the number of hypertrophic chondrocytes per column and height of the hypertrophic zones were significantly increased in RES-treated animals (Table 1). Moreover, the average size of the terminal hypertrophic chondrocyte was increased in the RES group while the E2 group was similar to controls (Table 1). In the E2 group, 2 animals out of 5 had fused growth plates and therefore all data are based on observations in 3 animals only.

**Figure 5:**
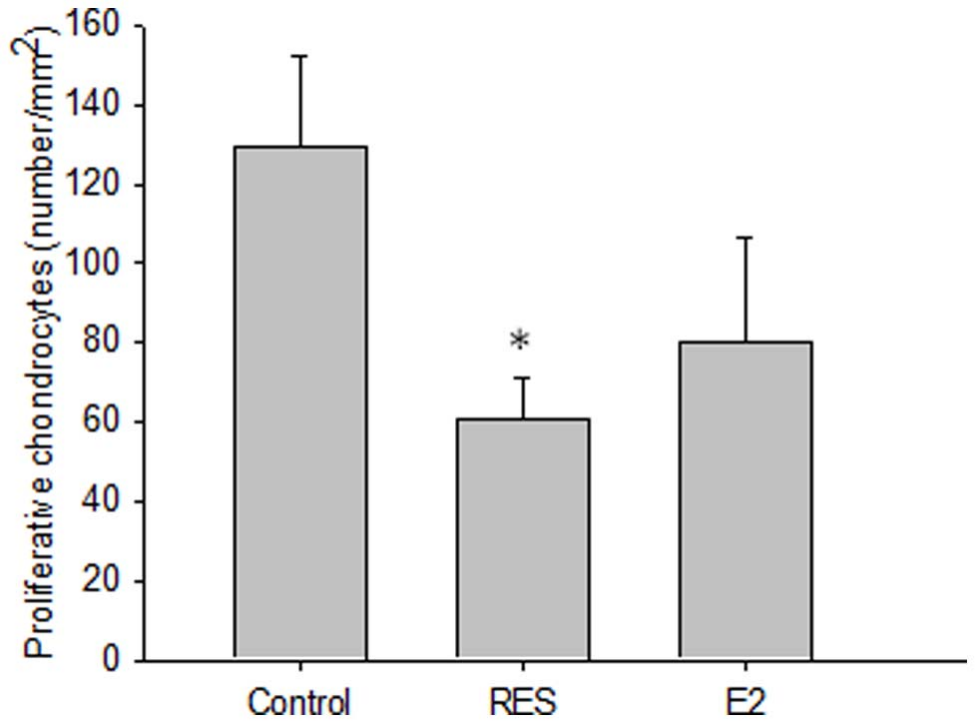
Number of BrdU positive chondrocytes per area in growth plates of vehicle, RES- and E2-treated OVX rabbits. ^*^p<0.05 vs. control.

Chondrocyte proliferation rate, as assessed by BrdU incorporation, was significantly lower in the RES group when compared with controls (Fig. 4 and 5). In E2-treated animals, there was a trend towards lower cell proliferation rate as well (Fig. 4 and 5). When studying the level of apoptosis, it was found to be similar in RES-treated OVX rabbits as in controls (0.2±0.1 vs. 0.2±0.1%) while significantly increased in E2 treated animals (1.1±0.4 vs. 0.2±0.1% in controls; p<0.05).

### Effects of RES on growth plate expression of VEGF and laminin in OVX rabbits

To determine whether growth plate vascularization was affected, the protein expressions of VEGF and laminin were examined by immunohistochemistry in the distal femur growth plate. We then found chondrocyte expression of VEGF to be clearly suppressed in RES-treated rabbits as compared to controls (265±54 vs. 626±50 VEGF positive cells/mm^2^; p<0.01) while in the E2 group VEGF expression was similar as in controls (632±153 vs. 626±50 positive cells/mm^2^; Fig. 4). Also the expression of laminin was decreased in the RES-treated group as compared to controls (17.7±0.6 vs. 27.4±1.04 positive cells/mm^2^; p<0.001; Figure S1). In contrast, laminin expression was elevated in E2 treated animals (44.4±0.8 vs. 27.4±1.04 positive cells/mm^2^ in control; p<0.001; Figure S1).

To investigate the expression of laminin at the chondro-osseous junction, the numbers of positive cells located within approximately 50 µm from the last hypertrophic chondrocytes towards the metaphyses were counted. Also here, the laminin expression was found to be lower in RES-treated animals as compared to controls (368±39 vs. 515±40 positive cells/mm^2^; p<0.05) while in E2-treated animals no difference from control was detected (458±57 vs. 515±40 positive cells/mm^2^). Due to the presence of bone bridges throughout the growth plates in E2-treated rabbits, laminin expression is however most likely underestimated in this group.

### Effects of RES on bone parameters and uterus weight

When compared to control rabbits, RES treatment did not significantly affect any bone parameters studied as assessed by pQCT after 7 or 10 weeks of treatment in OVX animals (data not shown). However, as expected, 7 weeks of E2 treatment significantly increased cortical bone density (1329±4.7 vs 1301±8.8 mg/cm^3^ in control; p<0.05). Uterus weight was significantly increased by E2, but at all time points and regardless of presence or absence of gonads unaffected by RES (Table 1).

### Effect of RES on osteoclast formation at the chondro-osseous junction

To verify if osteoclast formation was affected, TRAP staining was performed. We then found that RES had no significant effect on osteoclast formation while E2 treated animals had significantly lower number of osteoclasts at the chondro-osseous junction as compared with controls (Table 1).

### Effects of RES on the growth of cultured fetal rat metatarsal bones

**Figure 6:**
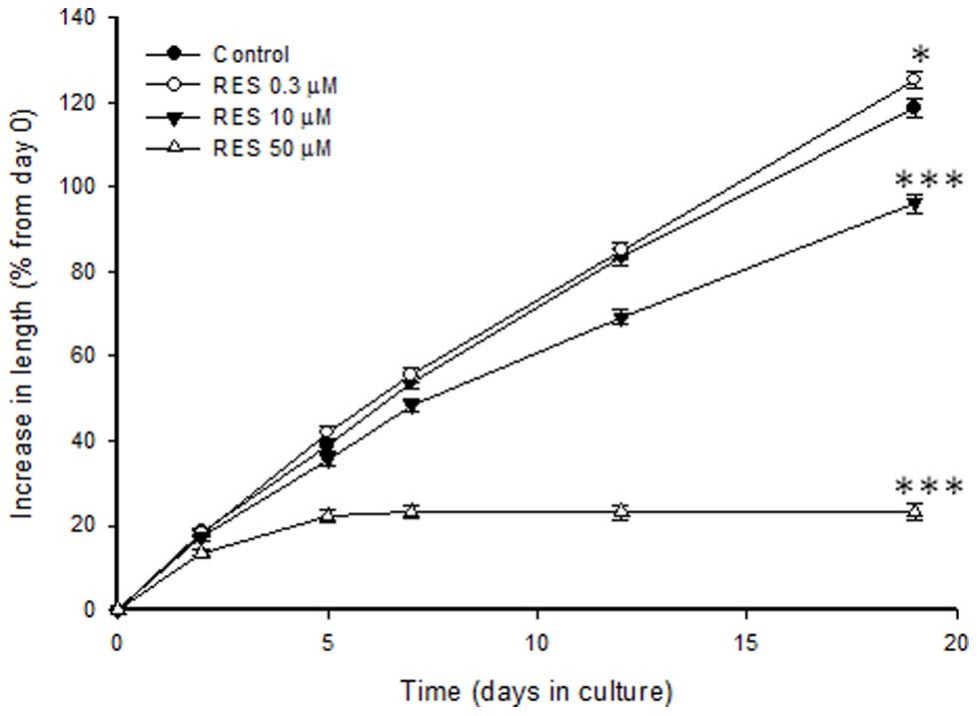
Dose-dependent effect of RES on longitudinal bone growth of fetal rat metatarsal bones cultured *ex vivo.* ^*^p<0.05 and ^***^p<0.001 vs. controls.

To study if the growth stimulatory effect of RES could be due to a local effect in the growth plate, we used a well-established model of fetal rat metatarsal bones cultured *ex vivo* [22]. Dose-response studies revealed that at 0.3 µM, RES-treated bones were significantly longer than control bones when measured after 19 days in culture (125.3±2.1% increase in length vs. 118.7±1.9% in controls; p<0.05). Of note, at very high concentrations (10 µM and 50 µM), RES suppressed bone growth (Fig. 6).

## Discussion

In the present study performed in both OVX and ovary-intact rabbits, we made the novel finding that RES significantly improves bone growth by delaying the process of epiphyseal fusion in female animals. Animals treated with RES had wider growth plates with enlarged resting zone, fewer proliferative chondrocytes, increased number and size of hypertrophic chondrocytes and markedly suppressed VEGF and laminin expression.

Indeed, RES increased bone growth and final length in pre-pubertal, ovary-intact female rabbits. We decided to start treating ovary-intact rabbits already at the age of 12 weeks to expose the rabbits to RES for a longer time period before sexual maturation. We observed a better growth rate for both vertebrae and tibiae which resulted in longer bones at the end. As the proximal tibia growth plate is the last epiphysis to fuse, it is therefore a crucial growth plate for determining final length [23].

To understand the underlying mechanism through which RES improves bone growth in ovary-intact rabbits, we also studied pubertal OVX rabbits which have wider growth plates and undergo epiphyseal fusion very slowly due to low estrogen levels. We considered E2 treatment as a positive control since it accelerates the time of growth plate fusion in all growth plates [23]. The model gives us the opportunity to measure the growth plate area longitudinally, to perform morphometrical analyses in growth plate cartilage and to study important para/autocrine factors involved in the process of epiphyseal fusion in a relatively wide growth plate.

It has earlier been shown that the timing of fusion varies between the different growth plates and that E2 accelerates this process in OVX rabbit where first the distal tibia growth plate fuses, then the distal femur and finally the proximal tibia growth plate [23]. In the present study, we found that RES had an opposite effect delaying growth plate fusion in all studied sites suggesting that those animals had more potential for further growth when compared with controls. At week 7, all animals had unfused proximal tibia growth plates and when the study was terminated at week 10, this growth plate was still open in all RES-treated rabbits while fused in 50% of control animals. Indeed, when measuring femur length after 10 weeks we confirmed such an effect, i.e. bones of RES-treated OVX rabbits were longer than those in control animals.

To investigate possible underlying mechanisms behind the growth stimulatory effect observed in the RES group, histomorphological studies were performed. We then found that RES treatment increases growth plate area, growth plate height, resting zone area, number of hypertrophic zone chondrocytes, and terminal hypertrophic chondrocyte cell size. The number of proliferative chondrocytes was not significantly affected by RES, albeit there was a trend towards a decrease, while the BrdU incorporation assay showed a significant decline in cell proliferation rate in RES treated animals. It is well known that chondrocyte proliferation, synthesis of extracellular matrix and cell enlargement all determine the rate of longitudinal bone growth. Our finding that RES can improve bone growth despite inhibiting chondrocyte proliferation may be explained by the fact that less than 10% of bone growth has been linked to cell proliferation while 60% results from chondrocyte hypertrophy and the rest from matrix deposition [29]. The observed increase in resting zone area in combination with suppressed chondrocyte proliferation rate altogether suggest that chondrocyte senescence is delayed by RES and that treated animals will have more capacity for further growth and increased final length compared to untreated animals. The facts that serum IGF-I levels were similar in RES-treated rabbits compared to controls and that RES had significant effects in an *ex vivo* model of cultured fetal rat metatarsal bones altogether supports a local growth plate action of RES.

It is important to point out that RES has a low bioavailability and rapid clearance when orally administered [12], [20], [30] and therefore relatively high doses must be given. We did not study a higher dose of RES based on the existing knowledge that high oral doses of RES (1 or 3 g/kg) are known to suppress food consumption and body weight gain in female rats and may even at the higher dose cause life threatening symptoms including kidney damage [20]. However, pharmacokinetic studies in healthy human subjects have not revealed any detrimental side effects caused by RES when administered in doses ranging between 0.3 mg up to 5 g per person yielding peak concentrations between 0.3 and 2.4 μmol/L [18], [31], [32].

*In vitro* studies have shown that RES at micro-molar concentrations has the capacity to bind to ERs [33] and inhibit aromatase activity [34]. Modulation of ER activity or inhibition of estrogen biosynthesis could theoretically be a possible mechanism through which RES is capable of promoting bone growth. We believe that the observed growth stimulatory effect of RES is not mediated through ER modulation because of its low bioavailability and, most importantly, its rapid clearance from the plasma which all together result in low serum concentration. However, we cannot ignore the possibility that the observed phenotype could be due to ERα antagonistic effects of RES.

Vascularization is an important process during epiphyseal fusion. Despite the growth plate being an avascular tissue, vascularization is important for substitution of cartilage tissue by bone tissue during endochondral ossification. It has been reported that RES inhibits tumor-induced neovascularization [35], [36] and wound healing [37]. Furthermore, it has been shown that the systemic administration of a soluble receptor chimeric protein Flt-(1-3)-IgG (VEGFR) to 24-day-old mice results in almost complete suppression of blood vessel invasion, concomitant with impaired trabecular bone formation and expansion of the hypertrophic chondrocyte zone [38]. This made us hypothesize that RES might modulate growth plate fusion by affecting the local expression of VEGF in the growth plate. We indeed found that VEGF expression was profoundly depleted in the growth plates of RES-treated animals while highly expressed in hypertrophic chondrocytes in control and E2-treated animals. Also the expression of another angiogenesis factor, laminin, well known to be expressed in the growth plate [39], was found to be decreased in the growth plate and at the chondro-osseous junction of RES-treated rabbits. Altogether, this suggests that RES has the capacity to suppress vascularization not only as previously described in pathological conditions but also in a normal tissue as the growth plate. We believe that the RES effect on bone growth and final length in this model could at least in part be explained by a decrease in VEGF and laminin expressions in growth plate cartilage. However, the fact that RES was also capable of stimulating metatarsal bone growth when cultured *ex vivo, i.e.* in the absence of blood supply, suggests that also other mechanisms than a down-regulation of angiogenesis factors contributes to the growth stimulatory effect of RES.

In summary, we found that RES delays growth plate fusion and thereby improves bone growth in female rabbits without having a systemic effect on the IGF-system. Based on our experimental data, we believe that RES is a safe medication and might have a potential to be used clinically to promote growth in short children by delaying growth plate fusion.

## Supporting Information

Figure S1 **Laminin expression in growth plates of vehicle, RES- and E2-treated animals.** No staining was detected when the primary antibody was omitted (negative control) while a strong cytoplasmic and extracellular matrix staining was detected when the primary antibody was applied to rabbit placenta (positive control). The area between the two red lines delineates the chondro-osseous junction. Bar  = 200 µm.
(TIF)
