# Supplementary figures and images for: Resveratrol Treatment Delays Growth Plate Fusion and Improves Bone Growth in Female Rabbits
Source: PLoS One. 2013 Jun 28;8(6):e67859. doi: 10.1371/journal.pone.0067859 (PMC3695926; doi:10.1371/journal.pone.0067859)

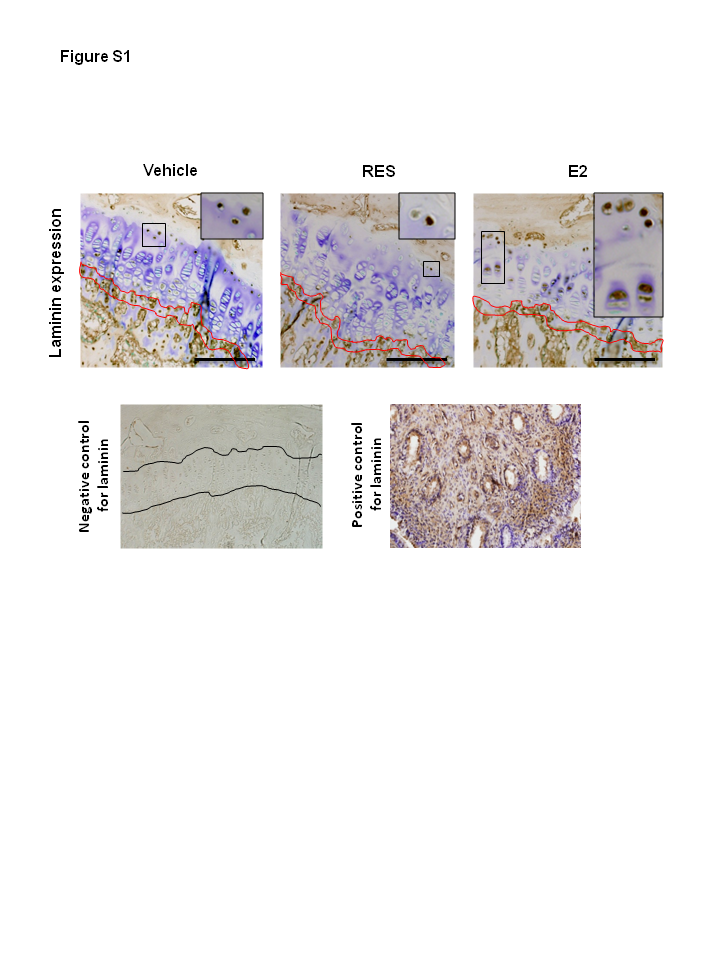

Supplement: Figure S1 — Laminin expression in growth plates of vehicle, RES- and E2-treated animals. No staining was detected when the primary antibody was omitted (negative control) while a strong cytoplasmic and extracellular matrix staining was detected when the primary antibody was applied to rabbit placenta (positive control). The area between the two red lines delineates the chondro-osseous junction. Bar = 200 µm. (TIF) [file pone.0067859.s001.tif]
